# Small Molecules Enhance Scaffold-Based Bone Grafts via Purinergic Receptor Signaling in Stem Cells

**DOI:** 10.3390/ijms19113601

**Published:** 2018-11-14

**Authors:** Patrick Frank Ottensmeyer, Markus Witzler, Margit Schulze, Edda Tobiasch

**Affiliations:** Department of Natural Sciences, Bonn-Rhine-Sieg University of Applied Sciences, D-53359 Rheinbach, Germany; patrick.ottensmeyer@h-brs.de (P.F.O.); markus.witzler@h-brs.de (M.W.); margit.schulze@h-brs.de (M.S.)

**Keywords:** purinergic receptors, mesenchymal stem cells, osteoclast, osteoblast, angiogenesis, bone, patent, scaffold, drug release

## Abstract

The need for bone grafts is high, due to age-related diseases, such as tumor resections, but also accidents, risky sports, and military conflicts. The gold standard for bone grafting is the use of autografts from the iliac crest, but the limited amount of accessible material demands new sources of bone replacement. The use of mesenchymal stem cells or their descendant cells, namely osteoblast, the bone-building cells and endothelial cells for angiogenesis, combined with artificial scaffolds, is a new approach. Mesenchymal stem cells (MSCs) can be obtained from the patient themselves, or from donors, as they barely cause an immune response in the recipient. However, MSCs never fully differentiate in vitro which might lead to unwanted effects in vivo. Interestingly, purinergic receptors can positively influence the differentiation of both osteoblasts and endothelial cells, using specific artificial ligands. An overview is given on purinergic receptor signaling in the most-needed cell types involved in bone metabolism—namely osteoblasts, osteoclasts, and endothelial cells. Furthermore, different types of scaffolds and their production methods will be elucidated. Finally, recent patents on scaffold materials, as wells as purinergic receptor-influencing molecules which might impact bone grafting, are discussed.

## 1. Introduction

Autologous bone grafts are considered the gold standard for therapies to overcome bone defects, due to their histocompatibility, osteoblastic cells, and osteoinductive factors, that are beneficial during grafting. Such bone grafts are obtained from iliac crest [1]. Next to the osteogenic properties, autografts induce their own vascularization. This enables the blood and nutrient supply of the graft, and increases implant survival [2]. However, there are also limitations, like donor site morbidity, due to an additional operation to obtain iliac crest bone grafts (ICBG), which could even result in the patient needing a wheelchair for up to six months. Last but not least, the amount of obtainable material is limited [3,4,5]. To overcome these restrictions, new graft sources that accumulate as waste products during surgery have been investigated for autologous bone grafting. An example of this is bone callus, that possesses an osteogenic potential due to contained osteoblasts, a porous structure, and osteoinductive factors, but the obtainable amounts are too small to fill the gap [6].

Since autografts do not cover the need for bone replacement, allografts are used [7,8]. Allografts are obtained from living or deceased donors, and stored in tissue banks after screening the medical and social history of the donors to exclude risk factors [9,10]. However, application of allografts is afflicted with an increased risk of rejection, due to the lack of perfect histocompatibility [11]. In addition, infectious disease transmission cannot be ruled out as well [12]. To overcome the risk of infections, diverse treatments of the graft are performed before transplantation, such as freeze-drying or anti-microbiological conditioning [13,14]. Good transplantation results can be achieved by pretreatment with gamma radiation, which is the most suitable method to sterilize biological tissues, including bone [15]. However, due to the limited availability of allografts, other options are still needed to supply bone for regenerative therapies. 

Xenografts derived from bovine or porcine sources are not able to induce osteogenesis of new bone because they must be decellularized before transplantation to avoid immune responses and, thus, rejection of the graft [16]. Despite the decellularization, xenografts showed comparable results in clinical studies compared to other bone graft materials [17]. To prevent immunological reactions and thus increase the use of xenografts, new approaches focus on the creation of humanized pigs with deleted pig and/or overexpressed human surface molecules but, also, modulation of anti-xenograft antibody expression prior to transplantation [18,19,20]. Nevertheless, to date, used xenografts can negatively interfere with the formation of new bone which leads to a decreased structure and stability of the bone [21]. Osteoinductivity in xenografts can be achieved by the incorporation of autologous cells, like mesenchymal stem cells (MSCs), that can be obtained from bone marrow or adipose tissue [22,23]. The embodiment of mesenchymal stem cells led to a higher integration of the graft to the bone, compared to the xeno-derived bone alone, since the cells are able to differentiate towards osteoblasts [24,25]. 

Next to xenografts, various artificial scaffold materials can be used for bone grafts as they do not carry the risk of host-mediated diseases, and can also be combined with autologous cells [26,27]. One of the main components of the bone is hydroxyapatite (HA), which is calcium phosphate and an interesting component also for synthetic bone grafts [28,29]. Synthetic scaffolds for bone grafts are available in adequate amounts, and can be shaped to fit in the cavity of the bone defect, for example, via 3D-printing, but the residues of monomers are often toxic, and could have adverse effects upon integration. Although synthetic bone grafts hold promising features, the risk of non-integration still remains higher compared to autologous grafts [30]. That is why current studies try to mimic the properties of autologous bone grafts. The addition of HA to scaffolds used for bone grafting enhances the differentiation of MSCs towards osteoblasts, presumably due to the similar environment as in bone, which can induce osteogenesis [31,32]. The combination of differentiated osteoblasts and osteoinductive factors or MSCs with bone grafts is of emerging interest, due to their ability to differentiate towards osteoblasts, which form the extracellular matrix of bone and lead to the generation of new bone, as well as fusion of the graft with the residual bone [33,34,35]. In fact, an increase in production of new bone can be observed in allografts loaded with MSCs compared to unloaded allografts or even autografts [36,37]. 

Next to osteoblasts, the bone resorbing osteoclasts, osteocytes, and vascular cells which initiate angiogenesis and, thus, blood supply of the graft, are of high interest [38,39]. A great benefit of MSCs is that they are also able to differentiate towards endothelial cells (ECs) and smooth muscle cells (SMCs) of the vascular system, which might be used to support vascular integration of the graft [40,41]. As not all stem cells differentiate in vitro, a promising possibility to increase and direct stem cell differentiation towards the desired cell type is preselection of the mesenchymal stem cells due to their different osteogenic potential or directed signaling via the purinergic receptors [42,43,44,45].

## 2. The Purinergic Receptor Family: Four Types Triggered by One Molecule and Its Derivatives

Purinergic receptors are the members of a highly conserved receptor family that is prevalent in most eukaryotes. They respond—as the name suggests—to ATP and its derivatives, with some receptors also responding to UTP and UDP [46,47]. Currently the purinergic receptors are classified in three subfamilies, namely P0 receptors, P1 receptors, and P2 receptors [47,48]. The latter is subdivided into the P2X receptors, which are ligand-gated ion channels, and the P2Y receptors, that are G protein-coupled receptors [49]. The naming of the subfamilies advises the binding molecule. P0 receptors are activated by adenine, the smallest derivative of ATP, whereas the P1 receptors respond to adenosine, and the P2 receptors to ATP and ADP [48].

### 2.1. The New Kid on the Block: P0 Receptors

The P0 receptors are barely investigated purinergic receptors. They were first described in 2002, by Bender and coworkers, as G protein-coupled receptors that were activated by adenine. They are divided in the rat adenine receptor and the mouse adenine receptor [50]. Upon activation, the receptors inhibit the adenylate cyclase via their coupled Gi protein [51]. Until now, the P0 receptor gene has been only identified in the rodents Chinese hamster, mouse, and rat. However, it is putatively expressed in the liver of pigs, and humans as well. To test this, binding studies with [H_3_] adenine and deazaadenine were performed on adult pig kidney cells and HEK293 cells, and the presence of adenine binding motifs was revealed on these cell types [50,52]. Tests with different synthetic ligands based on the structure of adenine rendered only a small number of ligands that can bind to the adenine receptor, and those are all agonists [53]. The function of P0 receptors and their role in bone development and homeostasis needs to be investigated. Also, more artificial ligands, especially antagonists, have to be developed for a better understanding of P0 receptor signaling.

### 2.2. Powered by Adenosine: P1 Receptors

There are four adenosine-responding P1 receptors called A1, A2A, A2B, and A3. The ligands of P1 receptors are adenosine and closely related structures, which show the high selectivity of these receptors [54]. P1 receptors are G protein-coupled receptors from the rhodopsin-like family, and consist of seven transmembrane domains and short N- and C-termini. A major target of the coupled G proteins is adenylate cyclase, which is inhibited by the Gi/o protein α-subunit of P1A1 and P1A3, and positively influenced by the Gs protein linked to P1A2A and P1A2B. However, depending on the coupled G protein, they can also affect other pathways [47]. P1 receptors can generally form homo- or heterodimers, but the function of this dimerization is mostly unknown. The only known homodimers are P1A1–P1A1 and P1A2A–P1A2A, which result in a stronger signaling upon ligand binding [55]. The investigated heterodimers consist of P1A1 and P1A2A, combined with other G protein-coupled receptors such as P2Y1 or P2Y2 with P1A1. Also, binding to other receptors, such as the dopamine D2 receptor, exists. The combination with P1A2A leads to a lower ability to respond to dopamine when an agonist for P1A2A is already bound to the heterodimer [54]. The P1 receptors are potential drug targets for a variety of diseases, such as antimetastatic therapies. The leading role of P1 receptors in bone formation and homeostasis leads to the suggestion to use artificial ligands for the treatment of bone diseases and grafting [56,57]. 

### 2.3. The Ion Channels: P2X Receptors

P2 receptors divide into two subgroups P2X and P2Y. The P2X receptors are a group of seven subunits, X1–7, that consist of two transmembrane domains and an interspacing extracellular polypeptide [58]. In general, P2 receptors are nonselective ion channels that open via a conformational change during the binding of extracellular ATP between the receptor subunits and enable mainly sodium, potassium, and calcium to pass the plasma membrane [58,59]. There are several homo- and heteromers of the P2X receptors that are known. An exception is the P2X6 receptor, which can only form heteromers, and the P2X7 receptor, which does not seem to be able to form heteromers, only homomers [60]. The varying phenotypes presumably have multiple functions next to ion channeling [61,62]. The most interesting P2X receptor for pharmaceutical applications seems to be the P2X7 receptor, that can initiate cell death in diverse cell types and is, therefore, a potential drug target with clinical trials pending [63,64,65]. The P2X7 receptor is expressed and regulated during differentiation of MSCs towards osteoblasts, which makes it also a promising target for bone regenerative therapies [66,67].

### 2.4. The Biggest Group: P2Y Receptors

P2Y receptors are G protein-coupled receptors that are either activated by purines, such as adenine nucleotide diphosphate or triphosphate, or by pyrimidines that are sometimes conjugated to sugars depending on the subtype. Eight subtypes of P2Ys have been identified so far, namely, P2Y1, P2Y2, P2Y4, P2Y6, P2Y11, P2Y12, P2Y13, and P2Y14 [47,68]. The missing numbers originate either from non-mammalian orthologues or receptors with a sequence homology to P2Y receptors, but without a functional response to nucleotides [68]. They are found in many different cell types. P2Y receptors consist of seven transmembrane domains that are connected via three intracellular, as well as three extracellular, amino acid loops [69]. By site-directed mutagenesis in P2Y1 and P2Y2, Jiang and colleagues discovered that the transmembrane domains 3, 6, and 7 are crucial for nucleotide binding to the receptors [70]. The conformation of the P2Y receptors can vary depending on the binding agonist and different G proteins coupled to the receptor. In the signaling pathway of P2Y11, the cAMP and inositol triphosphate concentrations increase when ATP binds to the receptor, but stay constant when UTP binds, which catalyzes calcium mobilization [49]. Due to the growing evidence of the participation of P2Y receptors in various diseases, such as Alzheimer’s disease or cardiovascular diseases, artificial ligands with high binding affinities are of interest for pharmaceutical applications [71,72,73]. Furthermore, the P2Y receptors are involved in osteoporosis, since they are influencing osteoclastogenesis [74]. 

Taken together, the role of purines and their receptors in the proliferation and differentiation of not only MSCs but, also, of hematopoietic stem cells (HSCs), is of great interest for bone grafting, due to the emerging use of stem cells in tissue replacement approaches, and the impact of purinergic receptors in the differentiation towards osteoclasts, osteoblasts, and vascular cells, respectively [45,59,66,75].

## 3. The Role of Purinergic Receptors during Osteogenesis and Angiogenesis 

**Figure 1:**
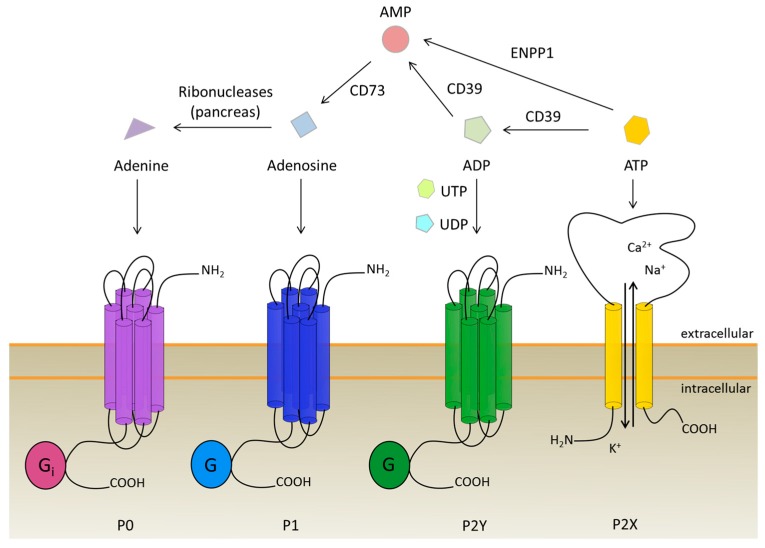
Purinergic receptors and their ligands with respect to ATP conversion to adenine. P0 receptors are activated by adenine, whereas P1 receptors are stimulated via adenosine binding. The P2 receptors are divided into ATP-dependent P2X ion channels and G protein-coupled P2Y receptors which are stimulated by ATP, ADP, UTP, UDP, and UDP-glucose. Degradation of ATP to adenosine is done by ectonucleotidases, namely CD39, CD73, and NPP1. Adenine is produced during the nucleotide recovery in the pancreas.

Similar to regular bone metabolism and homeostasis, bone grafts need to integrate into the existing bone via bone turnover processes which are regulated by bone resorbing osteoclasts and bone-building osteoblasts [76]. Here, the P1 receptors are of special interest, because these receptors and their natural ligand, adenosine, have an impact on osteoclast and osteoblast activity. The participation of purinergic receptors in bone homeostasis is not limited to P1 receptors; P2X and P2Y receptors and their ligands are also involved [77,78]. Changes between those receptor subfamilies are maintained by ecto- and endonucleotidases, such as CD73, CD39, or ectonucleotide pyrophosphatase/phosphodiesterase 1 (NPP1), that convert ATP to its derivatives and, therefore, influence osteogenesis (see Figure 1) [79,80]. Extracellular ATP, which is depleted by the nucleotidases, can be released from the stem cells, due to mechanical stimuli [81]. The role of purinergic receptor signaling in osteoblasts and osteoclasts will be described in the following paragraphs.

### 3.1. Insight into Purinergic Signaling during Osteogenesis

The adenosine receptors are expressed on osteoblasts as well as osteoclasts. While P1A1 is involved in HSC derivative differentiation towards osteoclasts, the role in bone formation is controversially discussed. D’Alimonte and colleagues showed an increase in osteogenic differentiation of dental pulp stem cells (DPSCs) depending on P1A1 and the Wnt signaling pathway, while Eijken published data on the inhibiting potential of the Wnt pathway in different stages of osteogenic differentiation [82,83,84]. The receptors, P1A2A and P1A2B, have an impact on new bone formation and osteogenic differentiation. By inhibiting adenosine uptake via dipyridamole, the number of osteoblasts increases in vivo in mice, depending on P1A2A signaling, implicating that the formation of new bone can be accelerated by addition of specific ligands for P1A2A [85]. Additionally, Costa and coworkers investigated the effect of adenosine receptors on MSC differentiation, which led to the conclusion that P1A2A stimulates osteoblast proliferation, but not differentiation, which is stimulated by P1A2B [86]. To prove this, Caroll and colleagues used mice lacking the P1A2B receptor, and detected a delay in fracture repair and lower expression of osteoblastic differentiation genes [87]. In line with these findings, investigations of rats with tibial bone defects resulted in an increase in bone repair due to stimulation of P1A2B [88]. Interestingly, Corciulo and colleagues discovered a higher calcified matrix production, collagen deposition, and alkaline phosphatase activity from mature osteoblasts in P1A2B knockout mice, although the number of osteoblasts decreased with the knockout [89]. The role of the P1A2B receptor on the function of mature osteoblasts was also investigated by Trincavelli and coworkers. They found that the receptor increases MSC differentiation towards osteoblasts, and also has a positive effect on the survival rate of mature osteoblasts [90]. The P1A3 receptor is not as well investigated as the other P1 receptors, due to its limited expression in most tissues. It seems to play no major role in bone homeostasis in in vitro experiments with rodent bone cells. P1A3 was only expressed in one of three osteoblast cell types and no effects on the formation of mineralized bone nodules were observed with either adenosine or 2-chloroadenosine. However, as both ligands are universal agonists for P1 receptors, the missing effect might be due to adverse effects of different receptors, as well [91]. Since, on the other hand, application of a specific agonist and antagonist for P1A3 revealed a positive effect on the proliferation of human primary osteoblast cells, in vitro, upon activation of P1A3, which makes it a possible drug target to induce osteoblast proliferation in bone grafts [86]. 

In addition to the adenosine receptors, the P2 receptors also determine the fate of MSCs during osteogenesis, as they are expressed on hMSCs in abundance, and several are regulated during osteogenesis [66,92]. Out of seven P2X receptor types, three seem to play a significant role in bone regeneration, namely the P2X1, P2X6, and P2X7 receptor, of which the P2X6 receptor has not yet been further investigated. The P2X1 receptor inhibits the formation of mineralized matrix via inhibition of the expression of alkaline phosphatase and, therefore, can be blocked by antagonists to increase bone mass [93]. The same is thought for the P2X7 receptor, but Manaka and colleagues found that the increased bone mineralization is independent from ALP expression [81]. The P2X7 receptor is involved in multiple pathways, such as apoptosis, Wnt, or MAPK signaling [94,95,96], which are key processes during osteogenesis. The influence on the p38/MAPK pathway was investigated via upstream P2X7 receptor inhibition. Specific P2X7 inhibitors were able to block shockwave-induced osteogenesis of hMSCs in vitro [94]. A supporting finding was made by Li and colleagues when they added BzATP, a P2X7 agonist, to differentiating hMSCs. In their experiments, the expression of osteogenic markers increased after activation of P2X7, which led to an increased phosphorylation of the other two MAP-kinases, ERK1/2 and JNK. This process can be reversed by the use of a specific antagonist [95]. The influence of the Wnt/β-catenin signaling pathway on osteogenic differentiation is also dependent on a stress reaction of the stem cells. During mechanical loading, ATP is released to the intracellular space, and activates the P2X7 receptor, which blocks the association of the active GSK-3β complex, which then leads to lysis of β-catenin and, also, induces a higher localization of β-catenin in the cytoplasm than in the nucleus. If P2X7 signaling went along with the binding of Wnt3a to the Frizzled receptor, then β-catenin accumulates in the nucleus and mediated the transcription of osteogenic genes [96]. The P2X7 receptor further increased osteogenesis in bone marrow-derived MSCs via the Rho-kinase axis by induction of formational changes of the plasma membrane [67,97]. While the cells undergoing zeiosis, the process of cell-blebbing during apoptosis, were from the femur, and the others from the calvaria, Zippel and colleagues suggested that beneficial effects of P2X7 are limited to precursor cells from the head region, as MSCs from lipoaspirates from other body regions have shown a negative effect of P2X7 overexpression on osteogenic differentiation [66]. Also, the addition of the P2X7 agonist reduced mineralization in mature osteoblasts [93]. The former might be due to a changing role of purinergic receptors during the differentiation state of the cells. A purinergic receptor that is beneficial during early osteogenesis can inhibit the process in late stages [98]. 

The P2Y receptors are all expressed in both MSCs and osteoblasts and, therefore, seem to play an important role in bone generation and bone homeostasis [67,99]. Early osteogenic differentiation, within the first one or two days, is inhibited by the activation of the P2Y2 receptor, after binding of its ligand UTP, via ERK1/2 signaling. The differentiation was increased by application of a specific antagonist during that time, which has a negative impact on the later stages of the process [100]. Generally, P2Y2 knockout mice have shown an increased bone volume [99]. On the other hand, they show a slightly decreased bone volume after eight weeks, and a decreased bone volume and bone strength 17 weeks after birth [101]. This finding underlines the changing roles of purinergic receptors during differentiation and bone homeostasis. A negative effect on osteogenic differentiation and bone homeostasis should be also expected in patients that use clopidogrel (Plavix^®^) for long-term treatment to reduce heart attacks and strokes. The P2Y12 receptor, which is inhibited by clopidogrel, seems to impact cell survival of both osteoblast precursor cells as well as mature osteoblasts, which decrease upon inhibition [102]. However, in another study, a positive effect of P2Y12 inhibition by clopidogrel on bone generation was shown, due to a decrease in osteoclast activity [103]. Also, the use of MSCs from P2Y13 receptor knockout mice showed a decrease of mineralized nodule formation via Alizarin Red S staining during in vitro differentiation [73]. Several studies are now focusing on the potential role of purinergic receptors in age-related bone diseases, like osteoporosis, e.g., single-nucleotide polymorphisms (SNPs) of purinergic receptors, that can be found in osteoporosis cohort studies to identify promising candidates for future therapeutic treatment [104,105].

P1A1 is needed for osteoclast function and, also, influences the differentiation of HSCs derivatives towards osteoclasts, whereas P1A2A and probably also P1A2B, inhibit the formation of osteoclasts and, in return, enhance bone regeneration and the differentiation of MSCs towards osteoblasts [106]. These findings show the presence of a balance between osteoblasts and osteoclasts, also in purinergic receptor signaling.

### 3.2. Insight into Purinergic Signaling During Osteoclastogenesis

A promising approach to impact on osteoclastogenesis and osteoclast function is adenosine receptors, because they are expressed on osteoclasts, as well as on osteoblasts and both cell types influence each other in a feedback loop. During osteoclastogenesis, osteoclast precursor cells fuse to multinucleated osteoclasts which are influenced by ATP and adenosine [107]. All adenosine receptor subtypes are upregulated during osteoclastogenesis [108]. Kara and colleagues specified P1A1 as the most important receptor subtype during in vitro differentiation of HSC derivatives [109]. This was also observed using a specific antagonist for P1A1 during osteoclastogenesis [110]. The receptor activator of nuclear factor κB(RANKL)-induced differentiation of HSC derivatives is inhibited. The P1A1 receptor blocks the association of tumor necrosis factor receptor-associated factor 6 (TRAF6) and transforming growth factor-β-activated kinase 1 (TAK1) to RANK, which are necessary for the activation of NF-κB and JNK [111]. The stimulation of P1A2A decreases NF-κB via macrophage colony-stimulating factor-1 (MCSF-1) and RANKL signaling during osteoclastogenesis, which makes specific P1A2A ligands possible drugs for osteoporosis treatment or site-specific inducers for implant integration [108]. Osteoclasts from P1A2B knockout mice showed a larger pit formation on dentin discs in vitro [89], which is in line with the findings that P1A2B stimulation inhibits the activation of NF-κB, ERK1/2, and p38 signaling and, thus, osteoclast cell-cell fusion [112]. This is also supported by the finding that a specific P1A2B agonist decreased the differentiation efficiency of HSC derivatives towards osteoclasts, as confirmed by TRAP staining in vitro [110]. However, recent studies did not unravel any effect of the P1A2A, A2B, or A3 receptor on the in vitro differentiation and function of mouse osteoclasts. The effect in this study was mediated by ATP and, therefore, P2 receptor-related [91]. 

It was reported that all P2X receptor subtypes, except P2X6 and all P2Y receptors, are expressed by osteoclasts, although some were only detectable in mouse [113]. A function was attributed to P2X4 as its activation induces a current in osteoclasts, potentiated by Zn^2+^ that stimulates bone resorption [114]. The role of P2X7 on osteoclast formation and function is discussed diverse, as a P2X7 knockout mouse model had slightly increased amounts of generated osteoclasts, but a decrease in bone resorption of the same cells [115]. On the other hand, Agrawal and Gartland reviewed the role of the P2X7 receptor in bone cell formation, and concluded that P2X7 activation is necessary for osteoclastogenesis, which is in line with the findings of Falzoni and colleagues that the P2X7 receptor is expressed during macrophage fusion [116,117]. The P2X7 receptor—also called suicide receptor—is involved in apoptosis, and regulates bone resorption via osteoclast survival. In contrast to the previously described P2X7 knockout study, another experiment indicated that, due to osteoclast survival in P2X7 knockout mice, the resorption of bone increases [118]. The fine-tuning of extracellular ATP levels, or the use of specific ligands for the P2X7 receptor, is pivotal for the survival of osteoclasts and, thus, their resorptive activity. The role of P2X receptors on inhibition of cathepsin K, which catalyzes the deconstruction of bone tissue and can be blocked by specific inhibitors, is not yet investigated, and might become important in the future [119].

From the group of P2Y receptors, the P2Y12 and P2Y14 receptors influence osteoclastogenesis. Mice which were treated with clopidogrel, a P2Y12 inhibitor, showed decreased resorptive activity of osteoclasts, although the number of osteoclasts itself did not change [103]. A potential role of P2Y12 in osteoclastogenesis could be deduced as Montelukast, a drug against asthma, decreased the number of osteoclasts in mice, which could be restored via the addition of ADP. The same effect was visible when a specific inhibitor of P2Y12 was used [120]. Since bone remodeling is needed for the integration of a graft into existing bone, it is notable that the deletion of the P2Y13 receptor in mice led to reduced bone turnover rates [121]. It can be postulated that a locally increased function of P2Y13 might increase bone turnover, site-directed at the bone graft integration site. Also, specific agonists for the P2Y14 receptor seem to increase bone turnover as the receptor stimulation increased the differentiation of osteoclast precursor cells in RANKL-induced osteoclastogenesis, and decreased this differentiation upon use of siRNA against P2Y14 [122]. 

**Figure 2:**
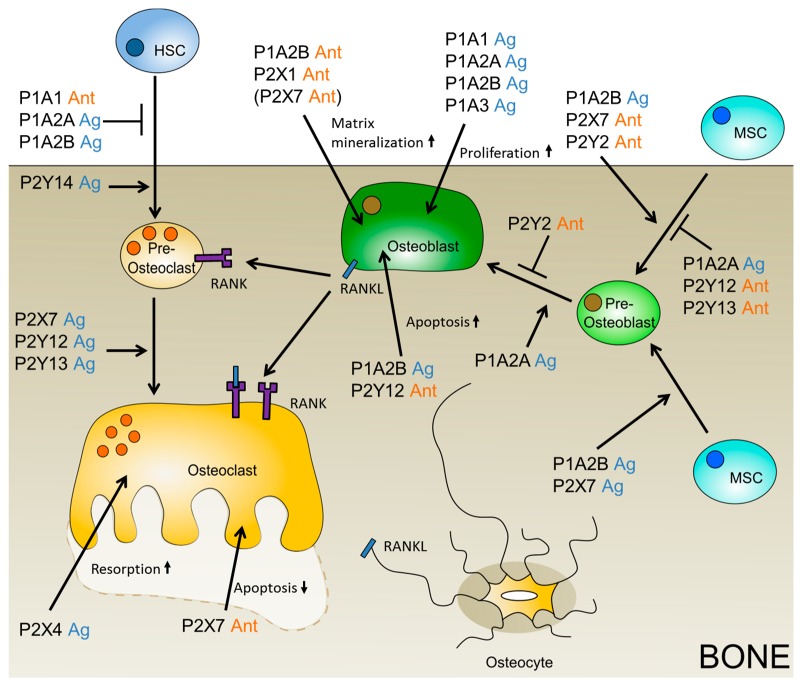
Putative purinergic receptor ligands which might positively influence bone grafting. The beneficial ligand is depicted, agonist (Ag) is shown in blue, and antagonist (Ant) in orange. If only the data on the negatively influencing ligand is available, then the putative positively influencing ligand is given in brackets.

Taken together, purinergic receptors are important mediators in bone homeostasis as they influence bone cell precursor cells during their differentiation towards their lineage, as well as regulate function and survival of the mature cells (see Figure 2). 

Another role of purinergic receptors and nucleotides that is central for successful bone grafting is angiogenesis, to ensure the supply of the bone graft with nutrients and oxygen and, thus, cell survival.

### 3.3. The Role of Purinergic Receptors during Angiogenesis with Respect to Artificial Bone Grafting

**Figure 3:**
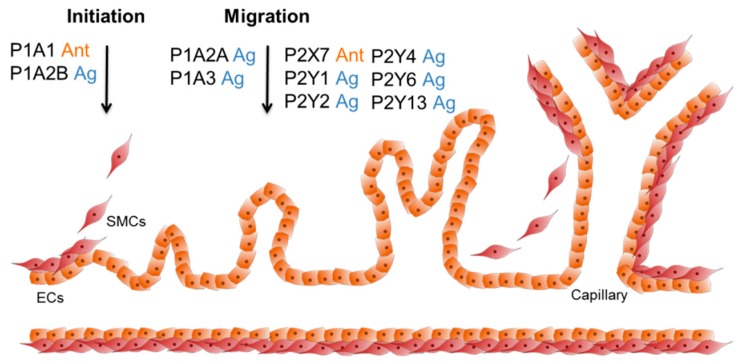
Purinergic receptor ligands which can influence angiogenesis. During the initiation phase of angiogenesis, the smooth muscle cells (SMCs) are released from the vascular tube. Next, the endothelial cells (ECs) start to migrate into the free space, and create new tubes and branches (sprouting). In the end, new SMCs cover the ECs and the process stops. P1A1 and P1A2B stimulate VEGF expression, which increases angiogenesis. The activation of P1A2A, P1A3, and P2Y1 enhances the migration of hEPCs and ECs. Additionally, P1A3 increases the expression of pro-angiogenic factors. The P2X7 receptor is accountable for EC survival and proliferation. P2Y2, P2Y4, and P2Y6 showed a pro-angiogenic effect upon Up4A signaling, and P2Y13 can increase angiogenesis by activation of Akt and ERK1/2.

Osteogenesis and angiogenesis are linked via the vascular endothelial growth factor (VEGF), which is secreted by multiple cell types in the vascular system, and stimulates the differentiation of MSCs towards osteoblasts, as well as towards endothelial cells [123,124]. The expression of VEGF is mediated by the P1A1 receptor, which upregulates VEGF release from monocytes, in vitro, upon stimulation [125]. The P1A1 receptor also activates angiogenesis in tumor blood vessels via VEGF-R2 [126]. The role of adenosine receptors in angiogenesis and endothelial cell sprouting was also investigated in CD73-deficient mice. CD73 is an ectonucleotidase that catalyzes the conversion of AMP to adenosine, the ligand of P1 receptors (see Figure 1). In the CD73-deficient mice, sprouting of ECs from blood vessels was decreased, whereas ECs from the lymphatic system were not affected [127]. Also, P1A2A and P1A3 activation enhanced the migration of human endothelial progenitor cells (EPCs) [128]. In contrast to this, Du and co-workers found that P1A2B had a positive effect on angiogenesis mediated by cAMP–PKA–CREB-driven VEGF production. The difference in findings might be explained by the use of different cells: the HMEC-1 cell line vs primary EPCs [129]. P1A3, furthermore, increases the density of microvessels, and promotes the expression of pro-angiogenic genes [126]. These findings make P1 agonists, which are more stable than adenosine, potential drugs for the induction of angiogenesis in bone-grafting strategies (see Figure 3). 

Since the P1 receptors are promising targets for angiogenesis, the question arises what influence the P2 receptors have on the formation of new blood vessels. In bovine cells of the two main blood vessel cell types, ECs and smooth muscle cells, all P2 receptors, except for P2X3 and P2Y6, were expressed in ECs, and all P2 receptors, except for P2X3, P2X7, P2Y6, and P2Y11, were expressed in SMCs [130]. In contrast to bovine SMCs, the expression of P2 receptors in human vascular SMCs is restricted to P2X1, P2X4, P2X7, and P2Y1, P2Y2, P2Y6, as well as P2Y11. In human vascular endothelial cells, the expression of P2Y1, P2Y2, P2Y6, and P2Y11 has been described [131]. However, only the given receptors and P2Y4 were tested. In other reports, also the expression of P2X7 receptor on rat endothelial cells was found, and a specific antagonist was further used to promote EPC proliferation [132]. However, the interplay with other pathways that e.g. inhibit EC apoptosis, must be considered as well [133,134,135]. Furthermore, P2Y1 is thought to account for healing processes of vascular injuries by activation of MAPK signaling pathways that contribute to the migration of ECs [136]. In the same study, they investigated the effect of P2Y12 and P2Y13 on EC migration by the application of specific ligands. However, the migration was not altered by signaling via the P2Y12 or P2Y13 receptor [136]. Lyubchenko and colleagues found that the P2Y13 receptor, together with the P2Y1 receptor, had the potential to increase angiogenesis via the Akt and ERK1/2 signaling pathway. This pathway influences ECs, as well as SMCs, and therefore offers a way to better-controlled angiogenesis [137,138]. A novel ligand for P2Y receptors, including P2Y2, P2Y4, and P2Y6, is uridine adenosine tetraphosphate (Up4A). It showed a pro-angiogenic effect in a co-culture model of human ECs, together with pericytes. This effect could be abolished by the addition of a P2Y6 inhibitor. This also makes the P2Y6 receptor an interesting target to induce angiogenesis (see Figure 3) [139]. 

Next to cells and angiogenesis, scaffolds are needed to provide a 3D matrix for cell attachment and formation of bone defect-specific grafts.

## 4. Strategies in Scaffold Manufacturing

Larger bone defects arising from tumor therapy or injuries often require implanted scaffolds to aid healing. Over the last two decades, there has been some kind of change in philosophy. While, back in the 1990s, most scaffolds were made of inert materials, nowadays, research has shifted to biocompatible materials that even improve cell attachment and bone formation. Over the years, numerous materials, both natural and synthetic, or even combinations thereof, have been investigated.

Recent research focuses mainly on synthetic ceramics, organic–inorganic hybrid materials, and polymer-based hydrogels. An ideal bone graft substitute, however, should mimic natural bone, in terms of properties and structure, as closely as possible, in order to promote wound healing and restore mechanical strength [140,141].

**Figure 4:**
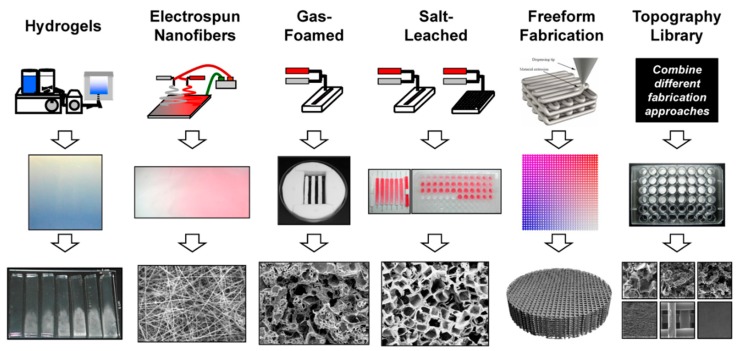
Different techniques for additive scaffold manufacturing (printed with permission of C. Simon Jr. [142]). First line: manufactured scaffolds include hydrogels, electrospun nanofibers, foams, and 3D structures obtained from salt leaching, freeform fabrication, and lithography. Second and third line: for each technique, the sample in macroscale is shown, together with a figure of the scaffold microstructure.

With the rise of affordable 3D printing devices, more and more studies include 3D-printed scaffolds of both organic and inorganic materials, and even composites. Other studies focus on hydrogels and hydrogel composites, on electrospinning, or approaches such as cements, pastes, and salt leaching processes. A schematic overview over manufacturing techniques is given in Figure 4 [142].

**Figure 5:**
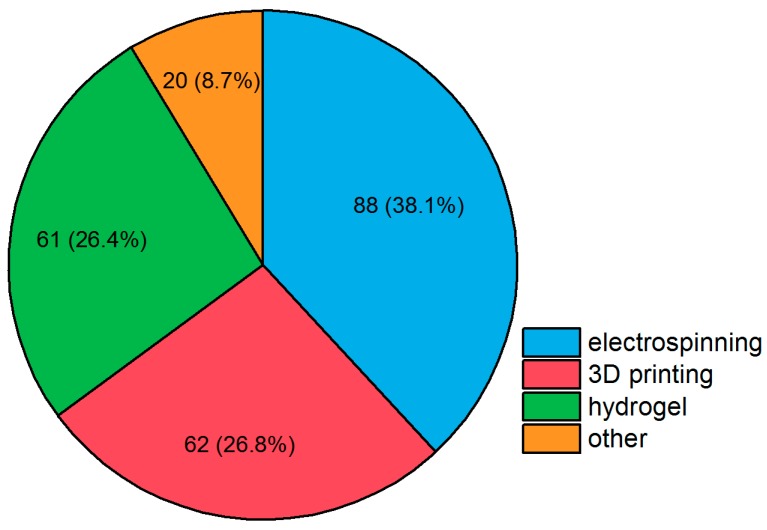
Publications between 01/2015 and 08/2018, partitioned by fabrication method of bone scaffold. Search parameters were publications with the terms “bone tissue engineering”, “scaffold”, and [fabrication method]. Search was performed on 21 August 2018, using NCBI PubMed. Reviews are excluded from the results. Possible differences can be due to rounding.

A PubMed database search (see Figure 5) shows that electrospinning makes up over a third, while 3D printing and hydrogel formation each make up approximately a fourth of current research. “Traditional” cements and all other approaches account for about 10% of all publications. The various techniques used in studies between 01/2015 and 08/2018 are presented more closely in the following sections focusing on selected publications.

### 4.1. 3D Printing

Three-dimensional printing is usually performed with easy-to-melt polymers, for an in vivo application, biopolymers and biocompatible polymers are under close investigation. Mixtures of poly-ϵ-caprolactone (PCL) and polyoxamine (Tetronic^®^) (in ratios of 100/0 to 80/20), with incorporated dexamethasone, were used in a melt-based scaffold formation process. In vitro testing showed good cell attachment and cell viability with the dexamethasone clearly promoting cell proliferation. However, this effect seems to be more dominant at earlier time points than at later stages [143]. Poly-ϵ-caprolactone was also used for 3D printing by Lee et al., together with a subsequent coating with polydopamine, which enhanced both wettability and grafting of recombinant human bone morphogenetic protein (rhBMP2) for better cell attachment and proliferation. Polymers are not the only material of interest in scaffold manufacturing by 3D printing: there is also focus on certain biocompatible ceramics [144]. Another group used mesoporous bioactive glass and tricalcium phosphate (MBG)-β-TCP composite scaffolds with MBG nanolayers in their printing. The MBG nanolayers resulted in a higher mechanical strength and better mineralization than β-TCP alone. Viability, cell activity in alkaline phosphatase (ALP) activity assay, and osteogenic gene expression of both rabbit bone marrow stromal MSCs and HUVECs (human umbilical vein endothelial cells), are enhanced in vitro. Additionally, a good in vivo bone formation in rabbit calvarial defects has been observed after four weeks [145]. Bose and colleagues reported a scaffold printed of β-TCP with incorporated Mg^2+^ and Si^4+^ ions, which showed sustained release of Mg and Si in phosphate-buffered saline (PBS) over 30 days. Incorporation of Mg/Si or varying the pore sizes of the scaffold (500–1000 µm) had no effect on mechanical strength compared to pure β-TCP, but scaffolds with additional Mg or Si showed increased blood vessel formation and accelerated bone formation in rat bone defect models. Apart from either polymer or ceramic printing, several groups work on polymer/ceramic composites fabricated via three-dimensional printing [146]. Murphy and coworkers produced a scaffold comprised of poly-ϵ-caprolactone and bioglass (~20 µm), which they imprinted with hMSCs in Matrigel^®^. The resulting scaffold had pores between 100 and 300 µm, which facilitated cell attachment. Additionally, in vivo degradation of bioglass leads to controlled and sustained release of a possible osteogenic drug [147]. Castilho and colleagues used TCP and alginate in two different approaches: TCP powder and alginate powder in mixed printing leads to scaffolds with areas consisting of either TCP or alginate. In contrast to this finding, printing a pure TCP scaffold and performing a vacuum infiltration with alginate solution led to porous, surface-coated scaffolds. Powder-mixed scaffolds performed better at mechanical testing and cell proliferation than vacuum-infiltrated, and a 2.5 wt % solution of alginate has been found to be better performing than pure TCP, or with higher alginate content [148]. The group of Park and colleagues also investigated β-TCP and poly-ϵ-caprolactone composites. They showed that higher TCP content (particles <20 µm) led to higher surface roughness, porosity, and wettability of the scaffold. Besides, it effectively promoted growth and osteogenic differentiation of D1 mouse MSCs in vitro [149]. Si-doped hydroxyapatite, and gelatin with incorporation of vancomycin, were also used for 3D printing. The resulting scaffold showed better biological behavior on pre-osteoblast MC3T3-E1 cell differentiation than pure hydroxyapatite (HA). The release of vancomycin in PBS follows first-order kinetics and provides antibiotics to the implant site over several hours and, hence, effectively inhibits bacterial growth [150]. Porous polylactide (PLA) scaffolds with pore sizes between 150 and 250 µm were 3D-printed by Grémare and coworkers, and showed good biocompatibility on human bone marrow stromal cells in MTT test, neutral red test, and live/dead staining [151]. Luo and colleagues prepared alginate/gelatin scaffolds with a coating of hydroxyapatite. The 3D-printed scaffolds were crosslinked with CaCl_2_, which also triggered mineralization. Mineralized scaffold showed higher mechanical strength, higher protein adsorption, and better proliferation and differentiation of rat bone marrow stem cells, in vitro, than unmineralized ones [152]. 

### 4.2. Electrospinning

A convenient way to produce fibrous and porous scaffolds, even at larger scale, is electrospinning. Polymer solutions are accelerated with high voltages; the resulting fiber is collected and subsequently dried. Rajzer and colleagues modified polylactide or poly-ϵ-caprolactone with the drug “osteogenon” prior to electrospinning. In the resulting fibers, the incorporated drug enhances bioactivity, mineralization, cell adhesion, and osteogenic differentiation, compared to non-modified fibers of the same materials [153]. The group of Xu and coworkers electrospun PLA into mats with a subsequent three-dimensional reprocessing into scaffolds by thermally induced nanofiber self-agglomeration (TISA). In short, the obtained mat is ground into nanofibers under liquid nitrogen. The nanofibers are then suspended, where they self-agglomerate at 55 °C. Lyophilization of the pellet results in a flexible, lightweight, and porous scaffold. Porosity is about 96% with pores up to ~300 µm. The fabricated scaffold promotes high cell viability of mouse MSC, and ossification in vivo [154]. The same group used their TISA method to prepare a PCL/PLA (4/1) blend with high porosity of 95%, and pores up to ~300 µm. Compared to neat PCL scaffolds, the blend has higher mechanical properties, shows enhanced hMSC viability and promotion of osteogenic differentiation in vitro, as well as better in vivo bone formation in mice [155]. Dhand and colleagues performed electrospinning of collagen containing catecholamines (norepinephrine or dopamine) and free Ca^2+^ ions, which led to partial polymerization and crosslinking of the nanofibers. The obtained mats were subsequently mineralized by treatment with (NH_4_)_2_CO_3_, in order to complete polymerization of the fibers and precipitation of CaCO_3_. The scaffold’s mechanical properties are similar to cancellous bone, and human fetal osteoblasts show enhanced cell adhesion, proliferation, and differentiation compared to neat collagen mats. With respect to the catecholamine, norepinephrine yields better results than dopamine [156]. The preparation of nanoHA/PHB (polyhydroxybutyrate) in thin layers and subsequent folding into porous 3D scaffolds has been reported by another group: bone marrow-derived MSCs show good adherence, proliferation, and osteogenic differentiation on scaffolds, in vitro. For further experiments, cells have been seeded onto the scaffolds, resulting in a bone graft which shows in vivo osteoid tissue and blood vessel ingrowth in mice [157]. Wu and coworkers reported nanographene oxide (nGO) made from starch, and starch dissolved in formic acid for electrospinning. Formic acid acts both as esterification agent and solvent for electrospinning. Scaffolds with nGO contents below 2.5 wt % show good biocompatibility, cell viability, and promote calcium phosphate mineralization of the fibers [158]. Shao and colleagues worked with a coaxial electrospun fiber containing a tussah silk fibroin/HA core, and a silk fibroin shell. Mechanical testing shows a 90-fold initial modulus, and 2-fold higher breaking stress than neat fibroin fibers. The resulting composite scaffolds facilitated adhesion and proliferation of osteoblast-like MG-63 cells, and showed higher biomineralization [159]. Additionally, not only electrospinning, but also centrifugal melt-spinning (CMS), should be worth mentioning: mats of poly-ϵ-caprolactone (PCL) were prepared via CMS and subsequently coated with collagen. Additionally, the coated mat had been crosslinked with polylactide (PLA) before MSC-laden alginate was printed onto the surface. The obtained scaffolds showed higher mechanical strength than neat PCL and MSC-laden scaffolds, and were more effective with regard to cell adhesion and proliferation than unladen scaffolds [160]. Cui and colleagues performed CMS with HA/PLA-co-glycolide, and subsequent supercritical CO_2_ treatment. They used no organic solvents, and received favorable structures for cell infiltration. The scaffolds showed improved compression strength, promotion of bone-derived MSC proliferation, and osteogenic differentiation. Also, enhanced in vivo osteogenic gene expression could be noted [161].

### 4.3. Hydrogels

Other approaches which require only standard laboratory equipment and no dedicated printing or spinning device use certain polymers and their ability to form hydrogels. Again, biocompatibility is required for scaffold preparation. Ma and colleagues mixed human-like collagen with diepoxyoctane-crosslinked pullulan for hydrogel formation. These hydrogels perform better than neat pullulan gels without collagen, with respect to biocompatibility, biodegradation, cell viability, and inflammatory response. A high molar weight of pullulan and higher collagen content both enhance mechanical strength of the scaffold, which showed no obvious degradation, in vivo, after 2 months [162]. Nguyen and colleagues encapsulated bone marrow-derived hMSC and HUVECs with alginate or collagen, and co-cultured them on either collagen or alginate hydrogels as scaffold material. Compared to alginate, collagen seems to be superior for vascularization [163]. Non-natural *N*-isopropylacrylamide hydrogels were investigated in both serum-containing and nonserum-containing media by Vo and colleagues. In both media, hydrogels show mineralization of calcium phosphates with a Ca/P ratio close to apatite, which indicates a possible bone formation in vivo [164]. Another group treated starch with citrate in order to gain a novel citrate starch hydrogel material. When placed in simulated body fluid, formation of an apatite layer shows potential biomineralization. Pure polymer hydrogels often lack mechanical strength and the ability to induce stem cell differentiation. However, hydrogels are used for encapsulation and guided release of osteoinductive drugs (e.g., growth factors). High mechanical strength is achieved using polymer–ceramic composites. Currently, numerous hybrid materials are being investigated [165]. Paris and coworkers investigated agarose/nanoHA hydrogels with respect to the incorporation of drugs. They mixed in the drug before gelling, performed the drop-in technique after gelling, and introduced the drug as chitosan capsules into the hydrogel. The combination of loaded capsules in loaded hydrogel provides the most consistent and sustained release [166].

In situ precipitation of hydroxyapatite showed homogenous dispersion of HA in the collagen or agarose hydrogel matrix. Porous scaffolds were fabricated by lyophilization, the addition of HA improves both thermal, and mechanical stability, as well as biocompatibility, cell viability, and proliferation of MG63 cells [167,168]. The group of Iwai and colleagues fabricated HA/agarose hydrogels using a soaking method. In short, hydrogels were alternately soaked into calcium- and phosphate-containing solutions. The composite was mixed with autologous bone and implanted to alveolar bone defects. Bone density increased, and neither infections nor increased inflammation were observed at the transplant site within one month [169]. Kolanthai and colleagues used a sol–gel technique of hydroxyapatite precipitation in agarose solution before gelling. Here, they investigated the dependency of scaffold properties on the solvent (water or ethanol). Water-based scaffolds show enhanced mechanical properties, wettability, drug loading, and hemocompatibility, compared to ethanol-based ones. Both support MC3T3 proliferation and mineralization in the absence of osteogenic differentiation supplements in media [170]. They also modified their method in order to gain HA nanorod/agarose composite powders by sol–gel in ethanol. Subsequent microwave heating increased crystallinity without destroying agarose content, and decreased particle size and zeta-potential, and further prolonged drug release. By contrast, heating the composite powder at 700 °C removes agarose and generates interconnected pores, but yields a lower total surface area [171].

### 4.4. Other Approaches

In addition to the abovementioned fabrication techniques, other methods are used in order to design scaffolds for bone tissue engineering.

Montesi and colleagues worked on functionalization of ceramics. HA-nanocrystals were functionalized with lactoferrin protein. The resulting scaffolds enhanced osteogenic differentiation of rabbit bone marrow MSC, and could potentially be used as injectable bone filling cement [172]. Abert and coworkers performed hot pressing fabrication of a composite consisting of PLA, β-TCP, and CaCO_3_ powders, and the obtained scaffolds show high bending strength, although swelling of the polymer matrix reduces strength and changes the volume and shape of the scaffold after incubation in aqueous media [173]. Biomimetic mineralized collagen (containing silica and apatite) release silicic acid in vitro, and stimulates osteogenic differentiation of mouse MSC and inhibits RANKL-mediated osteoclastogenesis, as reported by another group [174]. Thadavirul et al. demonstrated the fabrication of PCL, PCL-PHB, and PCL-PHBV (PHB-co-hydroxyvalerate) scaffolds, obtaining a porous structure by leaching of water-soluble salts. All polymer blends improve and support MC3T3-E1 cell attachment, proliferation, and mineralization, with PHBV being the most effective [175]. Poh and colleagues worked on melt extrusion blends consisting of PCL, PCL/bioglass, and PCL/Sr-bioglass, respectively. Neat PCL has also been coated with calcium phosphate (CaP) after extrusion. All blends have in vitro bioactivity, and support bone MSC proliferation. Only CaP-coated PCL scaffolds induce osteogenic gene upregulation in non-osteogenic media. All scaffolds showed in vivo in-growth of tissue, however, no mature bone formation has been observed [176]. Another group prepared composite scaffolds from calcium phosphate nanoparticles and collagen, tuning the porosity by incorporating ice particles that generated pores in the matrix after melting. Nanoparticles were loaded with dexamethasone, which was released into PBS over 20 days. Scaffolds exhibit good hMSC adhesion, viability, and proliferation in vitro. In vivo tests on athymic nude mice and histologic evaluation revealed increased bone and blood vessel formation [168]. A simple freeze-drying method for scaffold preparation has been established by the group of Tamburaci and coworkers: silica-containing diatomite was dispersed in chitosan solution, molded, and freeze-dried to obtain porous aerogels. Composites showed higher mechanical strength and improved biocompatibility with MG-63, Saos-2, and 3T3 cell lines [177].

## 5. Recent Patents and Clinical Trials in Bone Grafting via Scaffold Manufacturing

**Figure 6:**
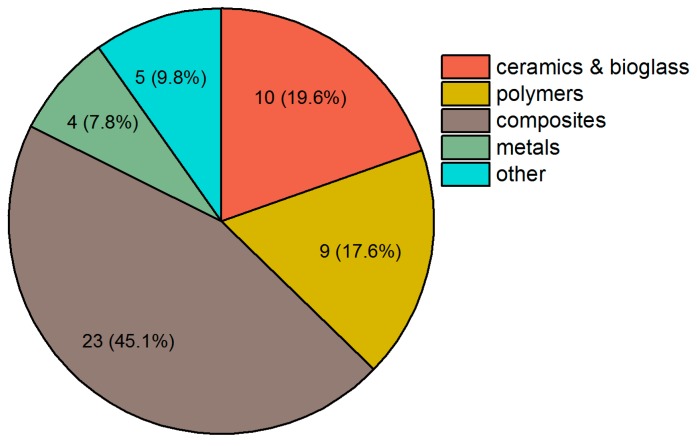
Patents between 01/2015 and 08/2018, partitioned by materials. Search was performed on Espacenet with the keywords “bone tissue engineering” and “scaffold” on August 21^st^, 2018. Composite materials had the most filed patents in the given period, followed by ceramics and bioglass, and polymers.

Between 2015 and 2018, several patents related to the field of bone tissue engineering using scaffolds have been filed. Among these, almost half of the inventors use polymer–ceramic composite systems as scaffold material. The different materials used in recent patents can be seen in Figure 6. The use of either polymers or ceramics is almost equally distributed, and only few patents deal with metal scaffolds or those from biological material, such as allografts or xenografts.

Those patents relying solely on ceramics are mainly using various sorts of calcium phosphates (including hydroxyapatite and tricalcium phosphate), although other ceramics, such as calcium carbonate, calcium silicate, zirconia, or silicon-substituted phosphate, have been reported to promote new bone formation or better implant–tissue connection [178]. For example, Schulz and coworkers invented a ceramic scaffold made of calcium phosphates or sulfates that exhibits a multiphasic resorption profile and can be used for increasing bone mineral density in osteoporosis patients [179].

Among polymer scaffolds, blends of different (bio)polymers are most commonly used. Biocompatible and biodegradable polymers, such as polysaccharides, silk fibroin, and PLA, are also widely investigated. Calabro and colleagues invented an implantable material that is crosslinked via hydroxyphenyl groups. Backbone of the material can be synthetic polycarboxylates or polyamines, or natural polymers like hyaluronan or aggrecan [180]. Cooper-White and others worked on a porous polymer blend of several natural and synthetic polymers that is designed for tissue engineering [181]. Das and colleagues invented a biocompatible and biodegradable polyethyleneglycol–polyurethane copolymer crosslinked with a triglyceride from castor oil that can be used for stem cell transplantation [182]. Saltzman and coworkers worked on biodegradable polylactic acid–glycolic acid nano- and microparticles for controlled delivery of proteins that could also find application in bone tissue engineering [183].

Creating composites from polymers and ceramics seems to be the most suitable approach for bone tissue engineering, due to the synergistic effects of both materials. Almost every patent featuring composites uses a calcium phosphate ceramic in order to encourage bone cell attachment. The broad variety results from polymers used for scaffold fabrication. Apart from few exceptions using PLA, polyvinyl alcohol (PVA), and PCL, most patents feature either collagen, silk fibroin, or natural polysaccharides, such as cellulose and chitosan. McKay and coworkers described implantable sheets made of calcium phosphate and collagen for bone regeneration. These sheets can be loaded with bone morphogenetic growth factors, and used as filling material or coating of other implant material, such as metal implants [184]. Müller and colleagues worked on a printable scaffold made of chitosan, calcium polyphosphates, and alginate. The composite is then further supplemented with a morphogenetically active ingredient [185]. 

The use of metals as scaffolds is less often part of current bone tissue engineering research due to their rigidity, however, a porous metal scaffold and a growth factor- and stem cell-coated trabecular metal scaffold have been described. Other bone substitutes from biomaterials also make up only a small part of recent patents in this field. Here, allografts specially coated for bone repair, and xenografts coated with platelet-rich plasma and marrow stromal cells are worth mentioning. Kipper and colleagues filed a patent for coating bone allografts with porous polymer layers or polyelectrolyte multilayers. The coating can also be used as a depot for the release of antibiotics, anti-inflammatory agents, growth factors, or other drugs [186].

In many cases, the material of the scaffold itself is not patented but, rather, the fabrication process or the scaffold’s geometry. Kumta and colleagues invented a scaffold made by additive manufacturing (e.g., 3D printing) with a lamellar structure [187]. Mechanical properties can then be further tuned by rotating the lamellae. Zhang and coworkers described a multi-step method for scaffold preparation and repair with pre-vascularization using mesenchymal stem cells. The scaffold can be made of alginate, several ceramics, such as hydroxyapatite, and other polymers [188]. Tayebi and coworkers invented a composite scaffold for bone regeneration with materials that have different rates of biodegradation and pore sizes. One of the materials further serves as a host for bone morphogenetic proteins which are to be released upon scaffold degradation [189].

Between 2015 and 2018, there are very few clinical trials to be found on either clinicaltrials.gov or clinicaltrialsregister.eu that deal specifically with scaffolds for bone healing. There are two active and recruiting studies that investigate scaffolds for bone regeneration: at the University of Kentucky, Lexington, KY, USA, two allografts (Osteocel Plus^TM^, NuVasive Inc. and alloOss^®^, ACE Surgical Supply Co., Inc.) are compared regarding their effectivity in healing maxillary sinus bone defects [190]. At the University of Jordan, Amman, Jordan, an MSC-seeded PLGA scaffold supplemented with human platelet lysate, for treatment of aneurysmal bone cysts, is currently investigated [191]. Not yet recruiting is a study from both Harvard University, Boston, MA, USA and Rambam health care campus, Haifa, Israel, comparing the effectiveness of a collagen scaffold (Ossix^®^ Volumax, Datum Biotech Ltd) to a freeze-dried bone allograft with collagen membrane, for alveolar ridge augmentation or dental implants [192].

## 6. Recent Patents on Purinergic Receptors That May Enhance Bone Grafting Strategies

Between 2008 and 2018, 31 patents, referring to purinergic receptors or nucleotidases in combination with bone or angiogenesis, were published. Since nucleotidases convert ATP to adenosine, which affects the P1 receptors, they must be considered as well. Referring to the patents published on nucleotidases (5) and P1 receptors (19), it seems that they play a greater role than P2 receptors (7) in actual patents.

### 6.1. Patents on Ectonucleotidases and P1 Receptors

NPP1 converts ATP directly to AMP and PPi (see Figure 1). The concentration of Pi and PPi is important for calcification of the bone matrix. Braddock and Albright invented a way to modulate NPP1 activity to regulate Pi/PPi proportion and, therefore, increase mineralization [193]. Furthermore, modulation of NPP1 activity influences P1 receptor signaling as AMP is degraded by CD73 to adenosine. Several groups developed compounds that are able to inhibit CD73 as a prevention for diseases not related to bone grafting [194,195,196]. Another way to block P1 receptor signaling are anti-CD73 antibodies, developed by a group from Innate Pharma [197]. These inhibitors and antibodies could be also used during bone grafting to enable integration of the bone by blockage of P1 receptors which could increase P2 signaling, such as P2X1, P2X7, or P2Y12, when it is beneficial for the differentiation. A step before CD73 is CD39, which converts ATP to ADP, and then to AMP (see Figure 1). Müller and coworkers developed a compound to inhibit the activity of CD39 in diseases as it influences the immune system during inflammation [196]. Benussan and colleagues established antibodies against CD39 to inhibit angiogenesis in tumors [198] and Jaschinski and coworkers developed an oligonucleotide sequence to inhibit CD39 expression [199]. Next to the tested effect on angiogenesis, these inhibitors could further improve P2 receptor signaling via modulation of CD39 expression and activity. A compound that influences the P1 receptors via inhibition was developed by Beatty and colleagues [200]. This compound could modulate bone turnover and graft integration due to the important role of P1 receptors on osteoclastogenesis and osteoclast function. 

An agonist to activate P1A2A receptor, as well as an antagonist to block P1A1 receptor stimulation, were developed by Cronstein and Medierto [201]. Stimulation of the P1A2A receptor is beneficial in the late stages of osteogenesis, whereas P1A1 activation increases osteoclastogenesis. These compounds, and an armada of antagonists and agonists for P1A2A for the treatment of neurodegenerative diseases, could theoretically be used to increase bone mass in a drug-repurposing approach [202,203,204,205,206,207,208,209]. Also, a broad range of P1A2B antagonists were patented during the last ten years that are most often intended for treatment of neurodegenerative diseases or for treating of ischemia. During ischemia, adenosine accumulates in the brain, but only the P1A1 receptor has a neuroprotective effect. The patented antagonists could be used to block P1A2B signaling during excessive adenosine concentrations in the brain [210,211,212,213]. The P1A3 receptor is beneficial for proliferation of mature osteoblast. Jacobson and colleagues developed an agonist for this receptor which, therefore, might be beneficial for bone grafting [214]. Other groups developed modulators or antagonists for the P1A3 receptor which could be used to increase the knowledge about this receptor in bone homeostasis and angiogenesis [215,216,217,218]. The high amount of patents for ectonucleotidases and P1 receptors shows their rising importance in therapeutic approaches against various diseases. The availability of agonists, antagonist, inhibitors, and modulators, therefore, might also influence bone grafting in the future.

### 6.2. Patents on P2 Receptors

Inhibition of nucleotidases affects the activation of P2X and P2Y receptors due to higher ATP and ADP concentrations. A patent focusing on P2X4/P2X7 signaling pathways was filed by Lee and Draganov and, like the patents of Benussan, concentrated on tumor growth and angiogenesis. Another approach used macrocyclic lactones, that could act as inhibitors for the involved P2 receptor pathways, such as P2X1, P2X7, P2Y12, P2Y13 during osteogenesis, and P2X7, P2Y1, P2Y2, P2Y4, P2Y6, and P2Y13 in angiogenesis [219,220]. P2X signaling can be further modulated by new compounds against P2X2/3 heterodimers, as well as against the P2X5 receptor, although their contribution to bone grafting and bone diseases is still under investigation [221,222,223]. A way to block P2X7 activity is via anti-P2X7 antibodies, which were developed by Barden and colleagues to inactivate non-functional P2X7 variants in cancer [224]. Benzamides, which are P2X7 inhibitors established by Kilburn and colleagues, could also be used to increase osteogenesis via P2X7 receptor modulation [225]. Angiogenesis can further be modulated in a negative way by a therapeutic approach using several different compounds, including P2Y1 and P2Y12 inhibitors, like clopidogrel, that could decrease endothelial cell migration into the bone graft and could be used to increase the knowledge about purinergic receptor-mediated angiogenesis [226]. 

## 7. Future Aspects of Purinergic Receptors and Their Ligands in Bone Grafting 

Taken together, purinergic signaling could play an important role during bone grafting. The use of specific ligands for purinergic receptors could enhance the integration of the graft into existing bone and its vascularization. However, the role of purinergic receptors is complex, as they play a role in a variety of different processes and in most cell types. Additionally, within such a process, the adverse effect of a receptor can turn into a beneficial one in later stages, as shown for osteogenic differentiation, where the receptor activation is biphasic [98]. 

Clinical studies are now focusing on the effect of SNPs in purinergic receptors related to different diseases in big cohort studies, which might narrow down the group to the key receptors. In this study, SNPs in purinergic receptors were found to increase the risk of osteoporosis due to a loss of function of the P2X7 receptor and the P2Y2 receptor [104,105]. Another clinical trial investigated the effect of caffeine, a general P1 antagonist, on the bone density of neonates. However, the results of this trial have not been published yet. A second study focusing on P1 receptors and bone mass investigated the effect of caffeine (and other risk factors, namely alcohol and smoking) on osteoporosis in men, but also for this trial, no results have been published yet, although the study already ended in 2001. However, in 2018, Goldman and colleagues published data on the effect of alcohol on bone mineral density, which was not related to purinergic receptors [227]. The role of the P2X5 receptor in bone diseases hints to a promising receptor to regulate bone homeostasis. In inflammatory bone loss due to osteoclast hyperactivity, the receptor was highly expressed and mediated IL-1β expression, which increased the formation of neutrophil granulocytes that lysed the extracellular matrix via cathepsin G [228]. The involvement of purinergic receptors in hard tissues, like tooth, is furthermore shown in a periodontitis mouse model. The inflammatory bone loss due to ligatureinduced periodontitis was decreased in P2X5^−/−^ mice [229]. Additionally, there is a patent focusing on this receptor and its inactivation from Darwish and colleagues. 

Taken together, the role of purinergic receptors in bone cells and angiogenesis must be elucidated in more detail, as it can change during differentiation, due to their influence on the various involved cell types, which can not only be negative or positive, but also biphasic, depending on the time point. The use of specific ligands at the different time points of bone grafting might increase the integration and survival chances of synthetic bone grafts combined with stem cells.

## Abbreviations

ALP	alkaline phosphatase
CMS	centrifugal melt spinning
DPSC	dental pulp stem cell
EC	endothelial cell
EPC	endothelial progenitor cell
HA	hydroxyapatite
HSC	hematopoietic stem cell
HUVECs	human umbilical vein endothelial cells
ICBG	iliac crest bone graft
MCSF-1	macrophage colony-stimulating factor-1
(MBG)-βTCP	mesoporous bioactive glass-β-tricalcium phosphate
MSC	mesenchymal stem cell
nGO	nanographene oxide
PBS	phosphate-buffered saline
PCL	poly-ϵ-caprolactone
PHB	polyhydroxybutyrate
PHBV	PHB-co-hydroxyvalerate
PLA	polylactide
PVA	polyvinyl alcohol
RANK	receptor activator of nuclear factor κB
RANKL	receptor activator of nuclear factor κB ligand
SMC	smooth muscle cell
SNP	single nucleotide polymorphism
TAK1	transforming growth factor-β-activated kinase 1
TISA	thermally induced nanofiber self-agglomeration
TRAF6	tumor necrosis factor receptor-associated factor 6
